# Covid 19 death analysis in Colombia

**DOI:** 10.15649/cuidarte.1528

**Published:** 2021-08-20

**Authors:** Hugo Alexander Rondón-Quintana, Carlos Alfonso Zafra-Mejía

**Affiliations:** 1 Universidad Distrital Francisco José de Caldas, Bogotá D.C., Colombia. E-mail: harondonq@udistrital.edu.co Universidad Distrital Francisco José de Caldas Universidad Distrital Francisco José de Caldas Bogotá D.C Colombia harondonq@udistrital.edu.co; 2 Universidad Distrital Francisco José de Caldas, Bogotá D.C., Colombia. E-mail: czafra@udistrital.edu.co Universidad Distrital Francisco José de Caldas Universidad Distrital Francisco José de Caldas Bogotá D.C Colombia czafra@udistrital.edu.co

**Keywords:** SARS-CoV-2, COVID-19, Colombia., SARS-CoV-2, COVID-19, Colombia., SARS-CoV-2, COVID-19, Colômbia

## Abstract

**Introduction:**

This article shows an analysis of the evolution up until date (May 4-2021), of official coronavirus cases statistics (CC) and the total number of deaths (TND) due to SARS-CoV-2 in Colombia. Additionally, said information is shown in correlation to other variables such as Case Fatality Rate (CFR), age range of persons, their typical reported co-morbidities and the cities where there has been highest concentration of cases.

**Materials and Methods:**

From March 16 2020 until today, information regarding the daily number of new confirmed cases (DNC) and daily confirmed deaths (DD) was registered in a database with the purpose of estimating the evolution of CC, TND and CFR. The age of deceased was also registered, as well as their gender, prior co-morbidities and city of death. The evolution of TND with the time of other countries were compared to that of Colombia. A mathematical equation that represents the epidemiological curve of TND evolution of different countries across time was defined.

**Results:**

In Colombia, the average age of people who die due to COVID-19 is of 69.5±14.7 years (median and mode of 71 and 80 years, respectively), and the virus is less lethal amongst a population under the age of 40. The greater part of deaths have taken place in people with prior co-morbidities and of the male gender.

**Conclusion:**

Most of the persons that have deceased are those of senior age, mainly with prior co-morbidities, and predominantly of male gender. Epidemiological peaks of COVID-19 are consistent with the rainy and winter seasons, and with the traditional epidemiological peaks of flu or influenza.

## Resumen

**Introducción:**

El artículo muestra un análisis de la evolución hasta la fecha (4 de mayo de 2021), de las estadísticas oficiales de casos de coronavirus (CC) y el número total de muertes (TND) por SARS-CoV-2 en Colombia. Dicha información se muestra en correlación con otras variables como son la tasa de casos fatales (CFR), el rango de edad de las personas, sus comorbilidades típicas reportadas y las ciudades donde ha habido mayor concentración de casos.

**Materiales y Métodos:**

En una base de datos fue registrada diariamente, desde el 16 de marzo de 2020 a la fecha, información concerniente al número diario de nuevos casos confirmados (DNC) y los fallecimientos diarios confirmados (DD). También se registró la edad de las personas fallecidas, así como su género, sus comorbilidades previas y ciudad de deceso. La evolución del TND con el tiempo de diferentes países fueron modelados matemáticamente y comparados con el de Colombia.

**Resultados:**

En Colombia, la edad promedio de las personas que mueren por COVID-19 es de 69.5±14.7 años (mediana y moda de 71 y 80 años, respectivamente) y el virus es menos letal en la población menor de 40 años. La mayor parte de las muertes se han producido en personas con comorbilidades previas y del género masculino.

**Conclusión:**

La mayoría de las personas fallecidas son personas de edad avanzada, principalmente con comorbilidades previas, y predominantemente de sexo masculino. Los picos epidemiológicos de COVID-19 son consistentes con las temporadas de lluvias e invierno, y con los picos epidemiológicos tradicionales de gripe o influenza.

## Resumo

**Introdução:**

O artigo mostra uma análise da evolução até o momento (4 de maio de 2021) das estatísticas oficiais de casos de coronavírus (CC) e do número total de mortes (TND) por SARS- -CoV-2 na Colômbia. Além disso, essa informação se mostra em correlação com outras variáveis como a taxa de casos fatais (CFR), a faixa etária das pessoas, suas comorbidades típicas relatadas e os municípios onde houve maior concentração de casos.

**Materiais e Métodos:**

Em um banco de dados, as informações referentes ao número diário de casos novos confirmados (DNC) e óbitos diários confirmados (DD) foram registradas diariamente, de 16 de março de 2020 até a data, para cálculo da evolução do CC, TND e CFR. Também foi registrada a idade do falecido, gênero, comorbidades anteriores e cidade do falecimento. A evolução do TND ao longo do tempo de outros países foi comparada com a da Colômbia. Foi definida uma equação matemática que representa a evolução do TND nos diferentes países ao longo do tempo.

**Resultados:**

Na Colômbia, a idade média das pessoas que morrem por COVID-19 é de 69.5±14.7 anos (mediana e moda de 71 e 80 anos, respectivamente), e o vírus é menos letal entre uma população com menos de 40 anos. A maior parte das mortes ocorreu em pessoas com comorbidades anteriores e do gênero masculino.

**Conclusão:**

A maioria das pessoas que faleceram são idosos, principalmente com comorbidades prévias, e predominantemente do sexo masculino. Os picos epidemiológicos de COVID-19 são consistentes com as estações chuvosa e de inverno e com os picos epidemiológicos tradicionais de gripe ou influenza.

## Introduction

Coronavirus SARS-CoV-2 (initially named 2019-nCoV and detected in Wuhan, China, on December 2019) is the responsible agent of the disease called COVID-19(1). Some of the important data on SARS-Cov-2 are: 1) at the initial stages, COVID-19 symptoms are mild, and a great number of persons may be asymptomatic and most people do not need hospital attention (around 81%)(2); ii) the reproduction number R0 (average number of new infections generated by an infected person in a totally unknowing population) is around 3.28(3); iii) the incubation period (time it takes from the moment of infection and the beginning of disease symptoms) is between a range of 4.75 days and 6.4 days(4), (5); iv) the series interval (period in which of two people with virus symptoms appear: the first person infecting the second, and the second, the infected one time of propagation) is between 2.6 and 7.5 days; v), CFR (Case Fatality Rate) is around 3%(6), and varies across countries with an average of 4.2 ± 3.8% (about half of countries had fatality rate >3.2% median)(7); vi) it has caused psychiatric implications(8) and it has created stress and anxiety, increasing the risk of generating mental diseases in the population (infected or not)(9); vii) COVID-19 increases its lethality exponentially with the increase of age(10) and the presence of comorbidities(11); viii) higher latitude and cold environment may be an additional risk factor for SARS-CoV-2 infection(12) and high temperature and humidity could reduce the transmission of COVID-19(13); ix) in some countries (e.g., Italy), the spread of COVID-19 is likely larger than officially reported(14).

Worldwide, up to the elaboration date of this article (May 4-2021) 153,676,825 confirmed persons have been infected with SARS-CoV-2, of which, more than 90.5 million have recovered and around 3,216,016 have died (an estimated current worldwide CFR is 2.09%). In Colombia, the number of confirmed infected persons by May 4 2021 is of 2,919,805, of which more than 2.74 million have recovered and y 75,627 have died (an estimated current average CFR of 2.59%).

This study had the main goal of organizing, presenting and analyzing the official information presented by the Ministerio de Salud [Health Ministry] (Colombia) through the Instituto Nacional de Salud (INS) and communications media in relation to the evolution of the deaths by COVID-19 in Colombia. The information presented was obtained from March 16 2020 until this date (May 4-2021).

## Materials and Methods

Daily information collected from March 16 2020 is official and comes from the Colombian Ministerio de Salud [Health Ministry]. This information was compared on a daily basis with that obtained from Colombian communications media and was input into a database. The information collected was the daily new confirmed cases (DNC) and daily confirmed deaths (DD). With this information was calculated the total number of coronavirus cases (CC), total number of deaths (TND) and CFR. Additionally, age, gender, prior comorbidities, and city of death were registered. For the case concerning deceased persons, the sample was of 75,577 (99.93% of total deaths registered up to this date). This information corresponds to days March 31, April 5 and April 9 2020 to May 4 2021.

On the other hand, the DNC and TND were registered for the following countries in order to compare with the Colombian case: Italy, Spain, France, United Kingdom and Germany. These countries were selected because of the impact generated in Colombia when its figures were registered in communications media. DNC and TND of Canada, Sweden, and Belgium were also registered. A mathematical equation that represents the epidemiological curve of TND evolution of Colombia across time was created.

The information registered in the database was organized and presented in Tables and Figures to facilitate its analysis. Besides, it has the purpose of being a source for consulting for other researchers in the field. The emphasis in analysis was mainly aspects related to deaths.

## Results

**Figure 1:**
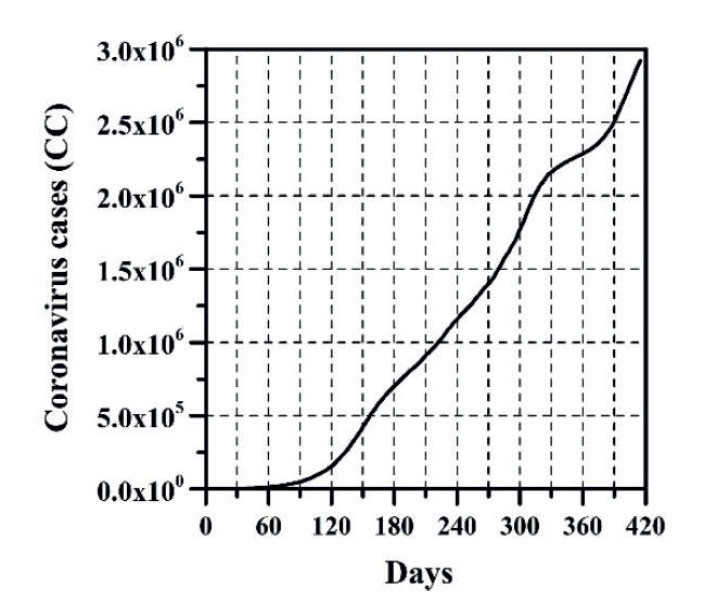
CC evolution per day

**Figure 2:**
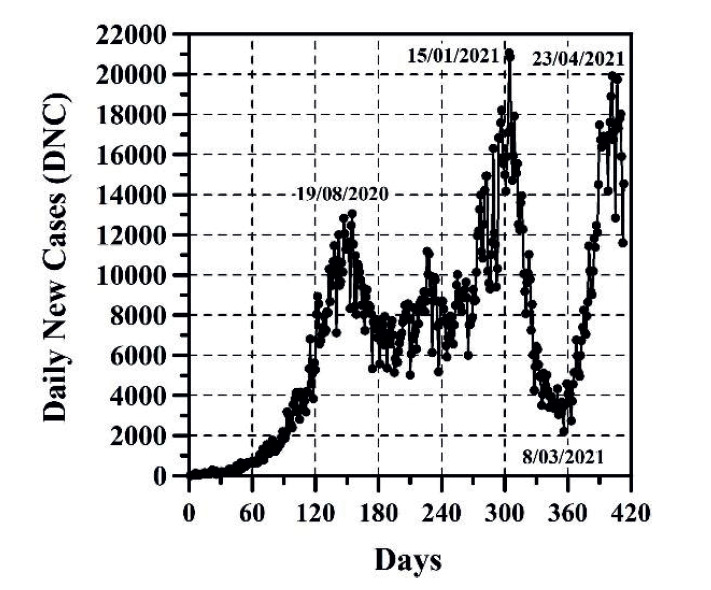
DNC evolution per day

**Figure 3:**
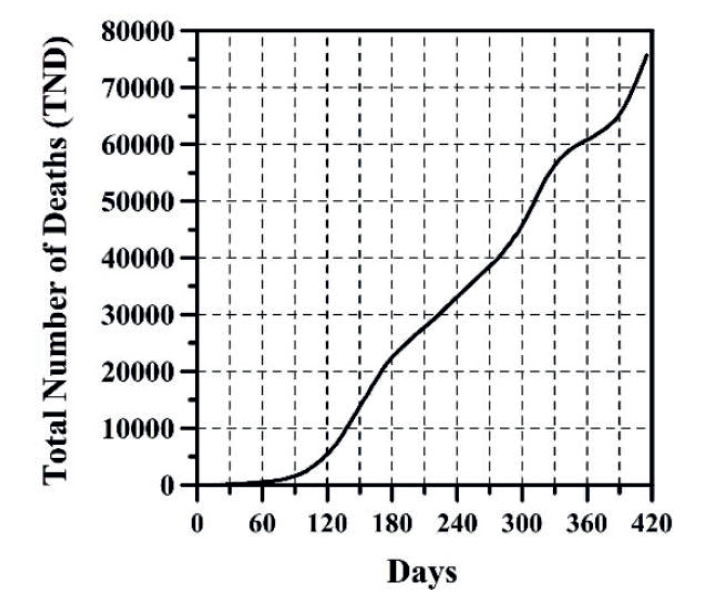
TND evolution per day

**Figure 4:**
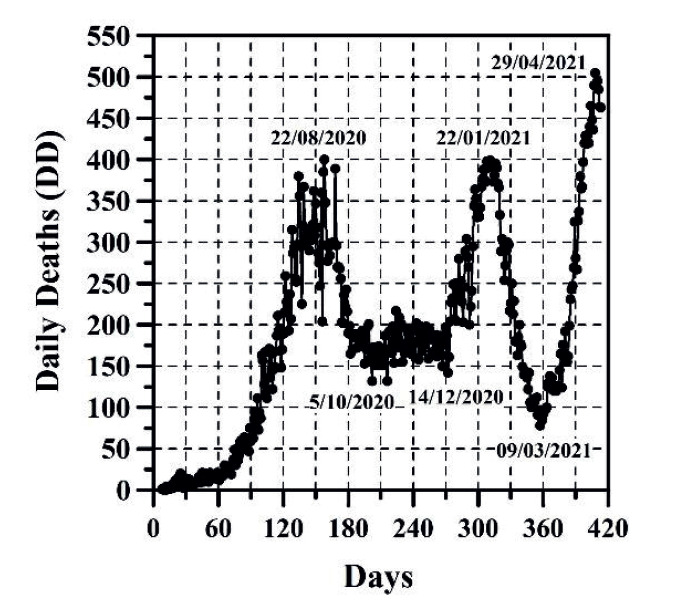
DD evolution per day

**Figure 5:**
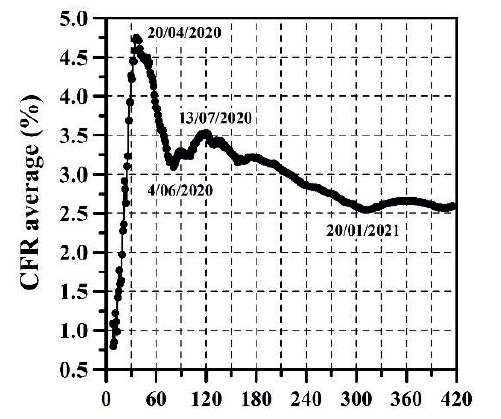
CFR average per day

**Figure 6:**
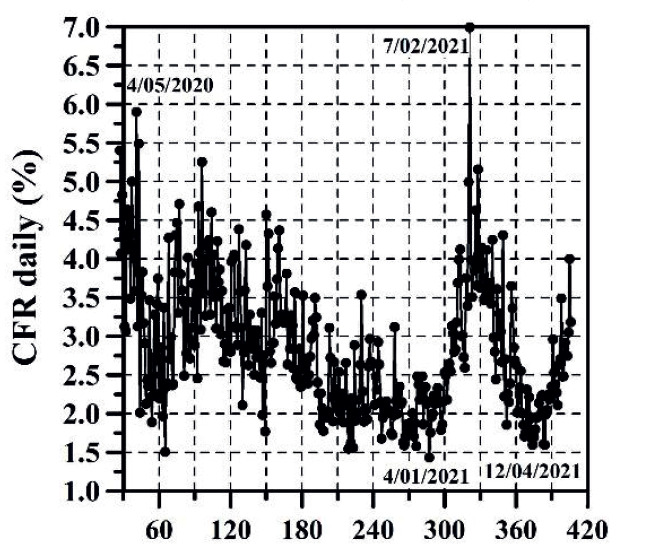
CFR daily per day

**Figure 7:**
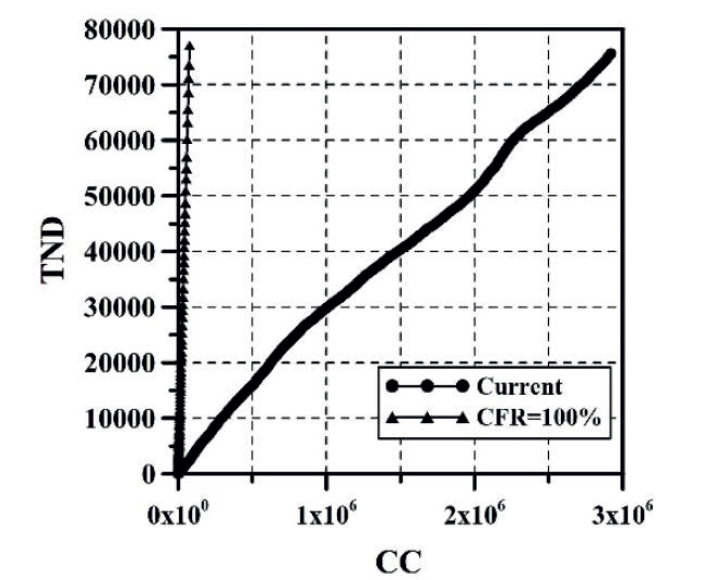
TND vs. CC

**Figure 8:**
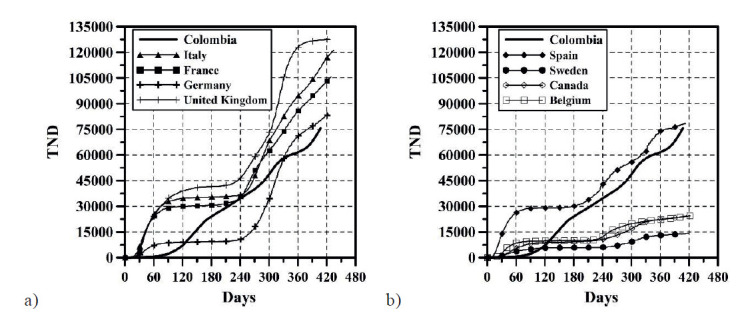
TND comparison with other countries. a) Italy, France, Germany and United Kingdom; b) Spain, Sweden, Canada and Belgium.

The growth of CC and the DNC are presented in Figures 1 and 2, respectively. The evolution of TND and DD can be observed in Figures 3 and 4, respectively. CFR average = TND/CC (%) and CFR daily = DD/DNC (%) are presented in Figures 5 and 6, respectively. The TND evolution in relation to CC is showed in Figure 7. Day 1 in all graphs corresponds to March 16 - 2020. The comparison of the Colombian TND with that of other countries is shown in Figure 8.

The mathematical representation of epidemiological curves of Colombia can be represented through equation (1) with an r2=0.99. Parameter *a* defines the maximum quasi-asymptote value of TND, while *b* and *c* moves the curve and defines its inflexion. *D* is the number of days.




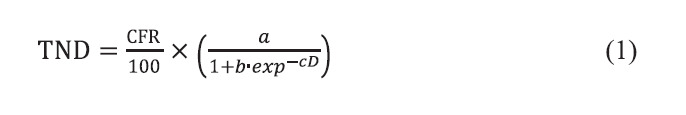

(1)



The distribution of CC and TND across ages is shown on Table 1.

**Table 1:** Distribution of CC, TND and CFR across age range

Age range (years)	CC	CC (%)	TND	TND (%)	TND accumulated (%)	CFR (%)
>100	508	0.02	163	0.22	0.22	32.1
90-99	12,996	0.45	4,393	5.81	6.03	33.8
80-89	62,779	2.15	16,177	21.40	27.43	25.8
70-79	133,469	4.57	20,488	27.11	54.54	15.4
60-69	257,676	8.83	17,475	23.12	77.66	6.8
50-59	405,879	13.90	9,643	12.76	90.42	2.4
40-49	478,028	16.37	4,316	5.71	96.13	0.90
30-39	656,298	22.48	1,956	2.59	98.72	0.30
20-29	619,418	21.21	775	1.03	99.75	0.125
10-19	197,993	6.78	102	0.13	99.88	0.052
0-9	94,761	3.25	89	0.12	100.00	0.094

**Figure 9:**
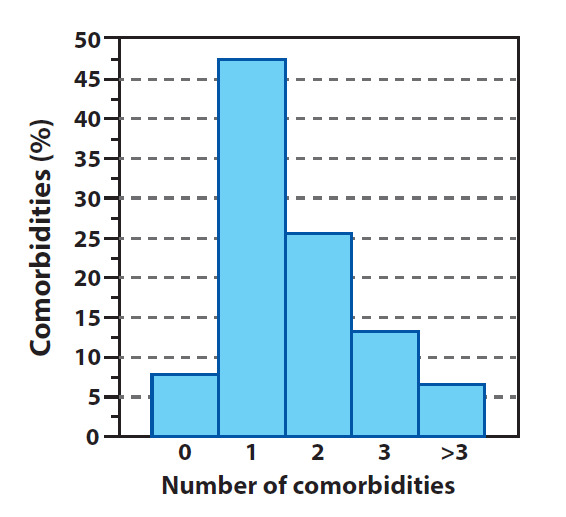
Comorbidity percentage

**Figure 10:**
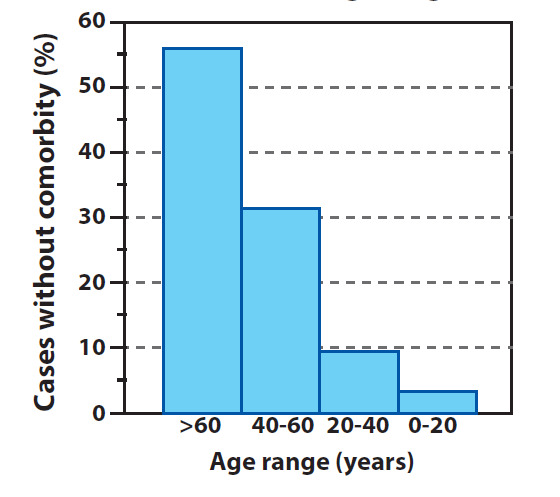
Percentage of deaths with no comorbidities across age range

The greatest part of TND corresponds to persons that have presented prior comorbidities. Unfortunately, the information provided by the Ministerio de Salud in relation to this topic, was very descriptive up until the day May 31 2020. From that date onward, a great portion of the deceased persons were presented in a manner which showed their comorbidity in a “in study” status, which did not allow for a rigorous quantification of those persons that formed a part of the TND which did not present comorbidities. Despite the above, the latest rigorous record of this information (until May 31 2020), showed that approximately 92.4% of deaths had been of persons that have had prior comorbidities distributed in the following manner (see Figure 9): 6.5% with more than 3 diseases, 13.1% with 3 diseases, 25.4% with 2 diseases and 47% with at least 1 disease. The rest of deaths (7.6%) have been of persons that had not shown comorbidities. Of this percentage of persons with no comorbidity deceased, the majority (56.3%) had ages above 60 years (see Figure 10) In other words, of 7.6% of deaths with no comorbidity 56.3% were of an advanced age. Very few persons with an age below 60 had died without prior diseases, and the trend was to lower this percentage as the person’s age was lower. Furthermore, up to May 31 2020, there have been no death reports of persons under 20 years of age and with no comorbidity (see Figure 10).

The main comorbidities present in people before death are listed on Table 2. These comorbidities were registered from March 16 2020 to May 4 2021.

**Table 2:** Typical comorbidities found in deceased

Comorbidities	%
Arterial hypertension (AHT)	27.8
Diabetes mellitus (DM)	15.8
kidney disease (KD)	9.6
Other cardiovasculardiseases (CVD)	9.4
Chronic obstructive pulmonary disease (COPD)	8.7
Obesity	8.4
Cancer	4.5
Brain and cerebrovascular diseases (B&C-D)	3.9
Hypothyroidism	2.3
Mental health	1.2
Autoimmune disease and Lupus	1.0
Malnutrition	0.71
Smoking	0.53
Asthma	0.53
Others pulmonary	0.53
Acquired immune deficiency syndrome (AIDS)	0.48
Hepatic cirrhosis	0.41
Anemia	0.34
Thrombosis	0.29
Prostatic hyperplasia	0.27
Tuberculosis	0.23
Arthritis and osteoarthritis	0.20
Apnea	0.16
Gastrointestinal (GI) bleeding	0.15
Other liver diseases	0.09
Dyslipidemia	0.07
Others	2.19

The distribution of TND in the country’s capitals is presented on Table 3, except for Soledad, which is a township belonging to the metropolitan area of Barranquilla and which CC and TND cases, unusually increased in some months.

**Table 3:** TND (%) in capital cities (*is not a capital city)

City	Population in 2019(15)	Population in 2019 (%)	TND (%)
Bogotá	7,412,566	15.36	21.19
Medellín	2,427,129	5.03	7.49
Cali	2,227,642	4.62	6.16
Barranquilla	1,206,319	2.50	5.63
Cúcuta	711,715	1.47	2.60
Soledad*	683,486	1.42	2.04
Bucaramanga	581,130	0.12	2.04
Ibagué	529,635	1.10	1.69
Santa marta	499,192	1.03	1.68
Montería	490,935	1.02	1.55
Cartagena	973,045	2.02	1.55
Neiva	357,392	0.74	1.41
Pasto	392,930	0.08	1.23
Pereira	467,269	0.97	1.23
Valledupar	490,075	1.02	1.07
Villavicencio	531,275	1.10	1.01
Manizales	434,403	0.90	0.87
Sincelejo	277,773	0.58	0.85
Florencia	168,346	0.35	0.65
Popayán	318,059	0.66	0.47
Riohacha	188,014	0.39	0.39
Leticia	48,144	0.10	0.30
Yopal	168,433	0.35	0.27
Tunja	172,548	0.36	0.26
Quibdó	129,237	0.27	0.17
Arauca	85,585	0.18	0.12
San Andrés	55,291	0.11	0.09
San José del Guaviare	52,815	0.11	0.05
Puerto Inírida	31,514	0.07	0.02
Mitú	29,850	0.01	0.02
Puerto Carreño	20,936	0.04	0.02
Other populations	26,999,330	55.95	35.89

## Discussion

In Colombia, projecting future CC, DNC, TND, DD and CFR is a complex task. Comparing with other countries, the evolution of these parameters has been different (Figure 8). In Colombia, for example, there are no long periods of time in which the DNC and DD have notably decreased (just as if it happened in other countries, mainly in summer; see stabilization of TND in other countries in Figure 8). For these reasons, TND evolution has always increase (sometimes linearly or exponentially with respect to time, see Figure 3). As can be observed (Figures 1-6), the distribution of these parameters in time has passed through five stages up until this date:

The first stage (from March 16 to end of August 2020), CC, DNC, TND, DD had an exponential growth. This first stage was due to the typical evolutionary growth of any epidemic. Around the first 30 days, from March 16 to April 15 2020, CFR average grew almost on a linear manner in time (an approximate rate of 0.16%/day) until reaching a value of 4.73% (Figure 5). From April 16 to the early June 2020, CC and TND continued growing exponentially, but the CFR average decreased in a linear manner from 4.7% to 3.15% approximately. Between June and August 2020, the CFR average varied between 3.15 and 3.5% approximately.

The second stage (from end of August to mid-September 2020), DNC and DD diminished. During this stage, good weather, closing borders, mandatory quarantine, among others public health measures could have helped reduce the virus speed transmission.

The third stage starts with constant DNC and DD values (from mid-September to midDecember 2020) and then increases (from mid-December 2020 to mid-January 2021). This behavior is perhaps due to i) the winter season began in Colombia; ii) quarantines were reduced and public health measures were more flexibles; iii) mid-December and midJanuary is the traditional vacation season in Colombia; iv) this stage coincides with one of the two traditional peaks of flu or influenza (September to December)(16).

In the fourth stage (from mid-January 2021 to early March 2021), DNC and DD decrease significantly. As was the case in Europe, the decrease in both parameters coincides with the summer season.

The increase of DNC and DD in the fifth stage (from early March 2021 until this date May 4 2021) agrees again with the rainy season and winter in Colombia. This stage coincides also with one of the two traditional peaks of flu or influenza (April to July)(16).

Given the above, predicting the evolution of CC, TND and CFR through a mathematical model is yet uncertain, because this depends on multiple variables such as: number and effectiveness of vaccines, capacity of making tests for detecting infected persons, strengthening the healthcare system, Government’s and people’s economic strength, mental health, social behavior, changes in Government strategies for tackling the pandemic, among others. So, in other words, predicting the arrival of the stage named by the National Government “Postpandemic” (evidenced when a great part of the population has been infected or vaccinated, the possibilities of infection are less and a relative sense of normality returns) is a complex endeavor. This is because any equation developed will represent an exponential evolutionary condition of variables CC and TND, which will tend to change in a future(17). Initially, every epidemic has exponential growth of CC and TND, however, this trend posteriorly changes to a relationship in which TND and CC begin to diminish, and the straight line shown on Figure 7 tends to become horizontal. Predicting D day, and the necessary CC required so that TND and CFR begin to descend is a task that depends on multiple variables shown before, which are not an object of this study.

A study reported by De la Hoz-Restrepo et al.(18) concluded that based on results obtained between March and July 25 2020, that Colombia has a lesser incidence of cases and deaths in comparison to other South American countries. However, in this date, the epidemiological curve in Colombia is just starting and conclusions were obtained based on less information. In a country with high poverty and inequality indicators(19), child malnutrition(20), violence(21) and unemployment(22), up to the date (May 4 2021), prolonged isolation and quarantines are not the best options. Additionally, when comparing behavior in other countries, and considering vaccine management, the population distribution, and the population number in these, it would seem that the end of the pandemic is yet far away from occurrence in Colombia. Perhaps an aspect that could reduce the evolution of TND in Colombia is its younger population in comparison to that of European countries. According to the DANE(23), Colombia’s population in 2019 was around 48.26 million inhabitants of which the distribution percentage across ages for men (48.8%) and women (51.2%) is of 22.6% for 0-14 years, 68.2% for 15-65 and 9.1% for older than 65. On average, European countries present a population distribution in which people above the age of 65 surpass 20%. In other words, the evolution of TND in Colombia could be mitigated by having a younger population.

The greatest number of deaths can be observed in advanced ages and is reduced with age. Slightly more than half of TND is reported by ages above 70 years (54.5%) and nearly – parts are reported for persons with an age above 60 years (77.7%). The other 22.3% is distributed across the following age ranges: 40-60 years (18.5%) 20-40 years (3.6%), under 20 years of age (0.25%). Additionally, the CFR decreases with age. The average range of the deceased is around 69.5 years of age with an approximate standard deviation of 14.7 years, median of 71 years and a mode of 80 years until April 30 2021 and changed to 70 years in the last 4 days. Approximately 37.4% of deaths have been of female gender (average age of death 70.3±15.0), the rest (62.6%) have been of male gender (average age of death 68.7±14.6). As with the other two types of coronavirus (SARS-CoV y MERS-CoV), apparently, older men with comorbidities are more susceptible to SARS-CoV-2 infections(6), (24), (25), because of the role played by the X chromosome and sexual hormones(26), among other possible causes. This is perhaps because, additionally, the greatest frequency of comorbidities and intense immune states observed in men(27), (28). Contrary to TND, a great part of the CC number is presented in young population. About a third part of CC (31.2%) is presented in persons with an age below 30, a little more than half (53.7%) is presented in persons with age under 40 and only 16.0% corresponds to those with an age above 60. Most part of CCs are found in the age range between 20 to 60 years (74%), which matches with the greatest part of the population in working age. It also tends to match with the population distribution of Colombia.

The data presented in Figures 9 and 10 is not unusual, several epidemiological studies have shown that persons with ages above 65 years and with prior comorbidities have a greater risk of developing severe complications and even dying because of other diseases classified as less lethal, such as influenza or flu, among others, as well as malnutrition, abuse, abandon, mental health etc.(29), (30), (31), (32). Additionally, in Colombia, risk factors for this type of population is two or three times higher in comparison to high income countries, where these types of problems increase amidst persons who live in economically and socially depressed areas, as well as geographically isolated areas, and when they suffer from arterial hypertension(33).

Apparently, the diseases that can most severely impact on the death of an infected person are (listed in higher recurrence to lower recurrence in Table 2): AHT, DM, KD, CVD, COPD, obesity, cancer, B&C-D, hypothyroidism, mental health, among others. These comorbidities are similar to those broadly reported for MERS-CoV infections(24),(34),(35),(36),(37). It is important to highlight that several researchers mention that obesity and/or smoking can be an indicator of negative prognosis for COVID-19(38),(39).

The highest TND values (a little more than a fifth of the TND, 21.2%) have been obtained in the country’s capital (Bogotá D.C.). This is because in close to 15.4% of the national population lives there and it has an estimated population density of 24,643 inhabitants/km2 (between 4 and 14 greater density compared to other cities that are important capitals on a national level). Barranquilla along with their adjacent township (Soledad), add up to almost 7.7% of TND. This could have occurred because of multiple factors such as: poverty, social indiscipline, possible high number of persons living in one housing unit, a considerable increase in the capacity of making tests for detecting infected, population (both add up to almost two million inhabitants) and their population density. On the other hand, almost half of the TND (43.1%) was obtained in the first five cities listed and a little more than half (50.5%) in the first nine cities listed. Around 2/3 parts of TND (64.1%) have been produced in the capital cities listed on Table 3 (capital cities count around 54.5% of the population). The rest, 35.9% is distributed as a minority across multiple cities and towns in the country.

## Conclusions

Based on the data presented, it is possible to conclude the following:

In Colombia, the greatest portion of deaths by COVID-19 have been people with ages above 65 years of age (average, standard deviation, median and mode of 69.5, 14.7, 71 and 80 years, respectively) and with prior comorbidities or diseases, such as AHT, DM, KD, CVD, COPD, obesity, cancer, B&C-D, hypothyroidism, mental health, AIDS, among others. Additionally, the risks of death increase among these persons if they are obese and/or smoke. CFR decreases markedly with age. On the other hand, men are more likely to die than women. In other words, this is a virus that have mainly affected a vulnerable population that has been fully identified, on which the greatest portion of strategies for fighting COVID-19 should focus: senior aged persons with prior comorbidities and predominantly of male gender. Contrary to the above, among young population and under 40, the virus is less lethal, despite that the number of infections surpasses half.

Predicting the future of Colombia and the evolution of CC and TND, as well as the exact date in which the stage named by the National Government as “Postpandemic” will occur is a complex task. This is because for the moment, the development of any equation that will represent an exponential evolutionary condition of CC and TND, which will tend to change in the future towards a condition in which they will begin to drop. Additionally, CC and TND will change in the future based on multiple variables such as the Government’s capacity and the capacity of several territorial entities in the country to make tests for detecting infecting people, number and effectiveness of vaccines, changes in strategy that can be executed by the Government in order to tackle the pandemic, the number of people in the country with an age above 60 years and that have comorbidities, investment and strength of the healthcare system, the Government’s and people’s economic strength, health status (physical and mental) and social behavior of people, among others. The growth of DNC, CC, DD and TND in Colombia has occurred in five stages. It is interesting to note that the epidemiological peaks of COVID-19 are consistent with the rainy and winter seasons, and with the traditional epidemiological peaks of flu or influenza. This behavior is also observed in the other countries analyzed.

For now, the highest TND values (21.2%) have been obtained in Bogotá D.C. The greatest part of TND (62.1%) is concentrated in the capital cities because they concentrate most of the population (54.5%). The rest (37.9%) is distributed as a minority across multiple cities and towns in the country.

Future studies must approach topics related to socio-economic. Public health, cultural, political and environmental impacts that have been generated and that will be generated by the pandemic in Colombia. These studies must be approached, by comparing them to those obtained in other countries. Additionally, there must be a comparison on the lethality of COVID-19 with relation to other diseases acknowledged as less lethal (e.g. Influenza or flu etc.) all of this in order to help the country in a future pandemic crisis.
